# Detection of movement-related cortical potentials based on subject-independent training

**DOI:** 10.1007/s11517-012-1018-1

**Published:** 2013-01-03

**Authors:** Imran Khan Niazi, Ning Jiang, Mads Jochumsen, Jørgen Feldbæk Nielsen, Kim Dremstrup, Dario Farina

**Affiliations:** 1 Department of Health Science and Technology, Center for Sensory-Motor Interaction, Aalborg University, Aalborg, Denmark; 2 Department of Neurorehabilitation Engineering, Bernstein Focus Neurotechnology Göttingen, Bernstein Center for Computational Neuroscience, University Medical Center Göttingen, Georg-August University, Von-Siebold-Str. 4, 37075 Göttingen, Germany; 3 Strategic Technology Management, Otto Bock HealthCare GmbH, Duderstadt, Germany; 4 Hammel Neurorehabilitation and Research Centre, Research Unit, Voldbyvej 15, 8450 Hammel, Denmark

**Keywords:** EEG signal processing, Movement-related cortical potential, Stroke rehabilitation, Motor imaginary, Subject training

## Abstract

To allow a routinely use of brain–computer interfaces (BCI), there is a need to reduce or completely eliminate the time-consuming part of the individualized training of the user. In this study, we investigate the possibility of avoiding the individual training phase in the detection of movement intention in asynchronous BCIs based on movement-related cortical potential (MRCP). EEG signals were recorded during ballistic ankle dorsiflexions executed (ME) or imagined (MI) by 20 healthy subjects, and attempted by five stroke subjects. These recordings were used to identify a template (as average over all subjects) for the initial negative phase of the MRCPs, after the application of an optimized spatial filtering used for pre-processing. Using this template, the detection accuracy (mean ± SD) calculated as true positive rate (estimated with leave-one-out procedure) for ME was 69 ± 21 and 58 ± 11 % on single trial basis for healthy and stroke subjects, respectively. This performance was similar to that obtained using an individual template for each subject, which led to accuracies of 71 ± 6 and 55 ± 12 % for healthy and stroke subjects, respectively. The detection accuracy for the MI data was 65 ± 22 % with the average template and 60 ± 13 % with the individual template. These results indicate the possibility of detecting movement intention without an individual training phase and without a significant loss in performance.

## Introduction

A brain–computer interface (BCI) provides an alternative communication channel for healthy or disabled users from their brains to external environment. A BCI system can detect changes of the state of mind from the on-going EEG and control an external device (e.g., text-entry system, prosthesis, and computer game). Since no peripheral nerves or muscles are involved in this process, BCI systems may be used as an assistive technology for patients with severe motor disabilities, such as stroke or locked-in patients [4].

In classic BCI approaches, the systems require extensive, sometimes frustrating, training by the subjects [1, 6]. Nevertheless, recently, the training time for classification of movement tasks has been shown to be reduced to <30 min [3, 14], without significant loss in performance with respect to longer training strategies [10]. Moreover, a subject-independent calibration (training) of a BCI system has been also proposed for a two-class classification task [7].

In recent years, BCI research has gained interest in the field of rehabilitation [4, 16]. A BCI system can be used either for controlling an assistive device to replace a lost motor function [e.g., functional electric stimulation (FES) or robotic limbs] [2, 17] or for inducing task-specific neuroplasticity for motor recovery (neuromodulatory effect) [5]. For neuromodulatory applications, there is a need to artificially establish a causal relation with short latency between the movement intention and stimulation of muscle afferents [8]. For this purpose, the movement intention has to be detected by a self-paced BCI system with short latency. Long individualized training is not desirable in these applications because it may interfere with the neuromodulatory effect of the main intervention phase.

In this study, we focused on the detection of movement intention and we contribute to the development of a self-paced BCI system that does not require an individual training. Moreover, since the number of EEG channels should be reduced for clinical BCI applications [7, 12], we also focus on a processing method using a single Laplacian derivation (based on optimized linear combination of input channels). The general concept that allows subject-independent training is similar to that used in previous studies [7, 10], although those studies addressed classification of different movements, whereas we focus on the detection of movement intention for self-paced BCI. To limit the latency of detection, we use movement-related cortical potentials (MRCPs) as the brain signals on which the system operates. The initial negative phase of the MRCP indeed precedes the movement by ~1.5 s. The subject-independent training paradigm for detection of MRCPs is tested on healthy and stroke subjects and compared the performance with the individualized training approach.

## Methods

### Subjects

Twenty healthy subjects (23.3 ± 5.2 years) and five stroke patients (44.2 ± 20.1 years, 4 males, 2 left side affected) participated in the experiments. Infraction was the cause of stroke for two patients whereas the others had been diagnosed with hemorrhage. On average, 54.2 ± 29 days from the stroke event had passed before the data collection sessions. Lesions were located by CT or MRI scans. Three patients had their right sides affected and were diagnosed with hemorrhage whereas the remaining two had the left side affected and were diagnosed with infarction. The degree of disability was evaluated by Functional Independence Measure (FIM^®^). The FIM is widely used in rehabilitation settings to assess the general level of disability of a patient. The score consists of 18 items e.g. self-care, Sphincters, mobility, locomotion, communication, psychological, cognitive functions. Each item is rated on a 7 point ordinal scale (minimum total score is 18, maximum total is 126 points). None of the healthy subjects had known sensory–motor deficiencies or any history of psychological disorders. All subjects gave their informed consent before participation and the procedures were approved by the local ethics committee of Nordjylland, Denmark (N-20100067).

### Procedures

The subjects were seated comfortably in a chair, with the right leg secured in a custom made fixture. A pair of surface EMG electrodes was mounted on the tibialis anterior (TA) muscle of the right side (dominant in all cases). Surface EMG signals were recorded in bipolar derivation, amplified with gain 1 k (healthy subjects: EMG-16 amplifier, OT Bioelettronica; stroke patients: BrainAmp EXG, Brain Products). The EMG signals were sampled at 1,000 Hz for healthy subjects and 2,500 Hz for the patients. Different amplification systems were used for healthy subjects and patients since patients were assessed in a clinical setting. The reference electrode was placed at the ankle. Monopolar EEG signals were recorded (EEG amplifiers, Nuamps Express, Neuroscan and BrainAmp DC, Brain Products for healthy subjects and stroke patients, respectively) from Ag/AgCl scalp electrodes (EC80, Easy cap) (healthy) and from an active electrode cap (actiCAP, Brain Products, Germany) (patients). The electrodes were located at the International 10–20 system locations FP1, F3, F4, FCz, Pz, P3, P4, C3, C4, and Cz. The right ear lobe was used as a reference and the ground electrode was placed at nasion.

Healthy subjects were instructed to perform ballistic ankle dorsiflexions, at random intervals. No external stimuli or cues were presented to the subjects for task executions. They were instructed to reach a torque level corresponding to 20–30 % of the maximum voluntary contraction (MVC). This procedure resulted in a fully self-paced set of executed movements (ME). In each experimental session, on average 2–3 runs of ~5 min duration each were recorded, with rest periods of 2–3 min in between. In each run, the subjects performed on average 15 trials.

The same healthy subjects also performed motor imagination (MI) with the same paradigm as described above on a separate day. In this experimental session, the subjects were asked to imagine the kinematics of ballistic ankle dorsiflexion without executing it. In this session, four runs of 5 min duration were performed. During the first two runs, the subjects performed the real movements, to develop their strategies of MI of ballistic ankle dorsiflextion. During the last two runs, they performed self-paced imaginary dorsiflexion. MI in this fully self-paced paradigm was identified with the press of a button by the subjects with their left thumb, approximately 2 s after the MI. The button press could potentially interfere with the detection of the MI task; however, for self-paced MI protocols, there is no standardized approach for the event labeling. Therefore, this study opted for this simple and relatively accurate event labeling technique.

To preliminarily validate the clinical applicability of the proposed approach, experiments were also performed on five hospitalized stroke patients. The patients were instructed to randomly attempt ballistic dorsiflexions of the right ankle, at a pace that they felt comfortable. No external stimuli or cues were presented to the subjects. A total of five runs of approximately 5 min duration were recorded with rest periods of 3–5 min in between.

The proposed detector is based on a template matching approach (matched filter; see Sect. “Signal analysis”). The template for the matched filter was built from the entire data set (excluding the test subject), therefore it was built without individual training (global detector, GD). This detector was compared with a detector built by a subject-specific calibration (individualized detector, ID). For the healthy group data sets, the training set for the individualized detector (ID) was built from the first one/two runs and tested on the remaining runs. For stroke patients, the initial two runs were used as training set and the last three runs were used as testing set for the ID. The global detector (GD) was tested in a similar fashion with a leave-one-out validation method. According to this procedure, the data of all but one subject were used for building the detector which was tested on the left-out subject. All the runs of the subject being tested were considered as testing data set (TST1) in one validation session. Moreover, for direct comparison with the ID approach, the GD was also tested for the testing subject only on the run(s) used for testing the ID (TST2-GD), such that the testing sets of TST2-GD were identical to the respective ID cases. In this way, the testing was executed on exactly the same data for the ID and the GD. For the MI data, separate templates were built for each session. For this purpose, the self-paced real movement (run 1 and 2) were used as training data sets, whereas the last two runs of self-paced MI were used as testing data sets for ID, as done in Ref. [14]. For GD, all the real movement runs of all the subjects, except the one being tested, were used as training set and the MI runs of the subject being tested were used as testing data set.

### Signal analysis

**Fig. 1:**
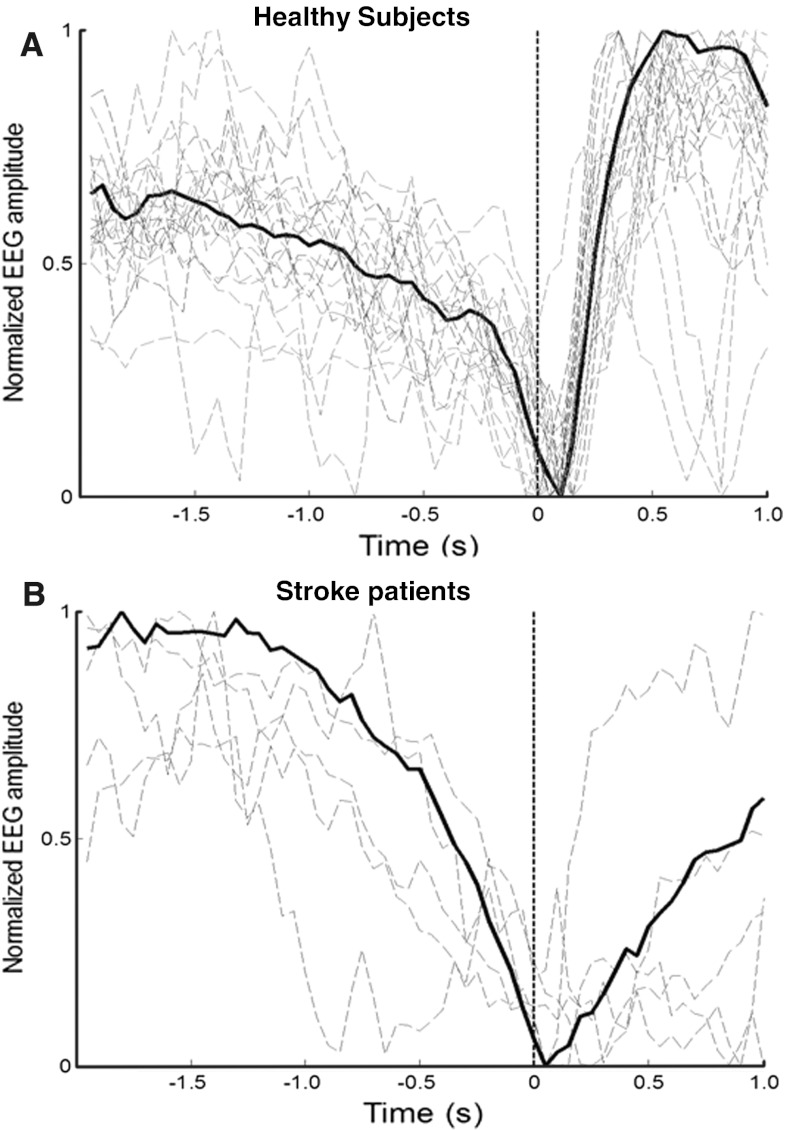
Normalized individual and global MRCP. MRCP obtained with OSF for healthy and stroke subjects. The *solid line* shows the average across all subjects. The *dashed lines* show the individual MRCPs. The initial negative phase of the MRCP (*part before the* *vertical dashed line*) was used as template for the matched filter

The EEG signals were band-pass filtered from 0.05 to 10 Hz, and then down-sampled to 20 Hz. The details of the spatial filtering and detector algorithm are presented in Ref. [14], where an individualized approach was used. In summary, the analysis was divided in two steps: MRCP template extraction and detection. First, the coefficients for an optimized spatial filter (OSF) were computed to maximize the signal-to-noise ratio (SNR). The initial negative phase of the MRCPs (from the start of the depression phase to its peak negativity, as illustrated in Fig. 1) in the spatially-filtered channel of the training data was used as template. The extracted MRCP template was used to detect movement intentions in the test data set by a matched filter. Before the detection of movement in the test set, a receiver operating characteristic (ROC) curve was obtained by varying the detection threshold. The threshold was selected at the knee of the ROC so that a balance between true positive rate (TPR) and number of false positives (FPs) could be obtained. The detector decision was based on a 2-s sliding window, with 200 ms shift.

A movement was identified in the EEG traces of the test data set when two of the three consecutive windows crossed the threshold corresponding to a desired false alarm probability. The following performance parameters were calculated: TPR (%), FPs per minute, and latencies (detection time with respect to the onset of the events). For the calculation of the latency, the reference movement onset (event) was estimated as the time instant when the rectified EMG signal amplitude crossed a threshold equal to one-tenth of its maximum during the ME. For stroke patients, the residual EMG signal was used in the same way as in healthy subjects to mark an event. For the MI data of healthy subjects, the latency could not be computed. Epochs with EOG activity exceeding 125 μV were discarded.

Paired *t* tests were applied to investigate the potential differences in the performance indexes and latencies between TST1 and ID and between TST2 and ID with TPR, in both the healthy and stroke subject group.

## Results

**Fig. 2:**
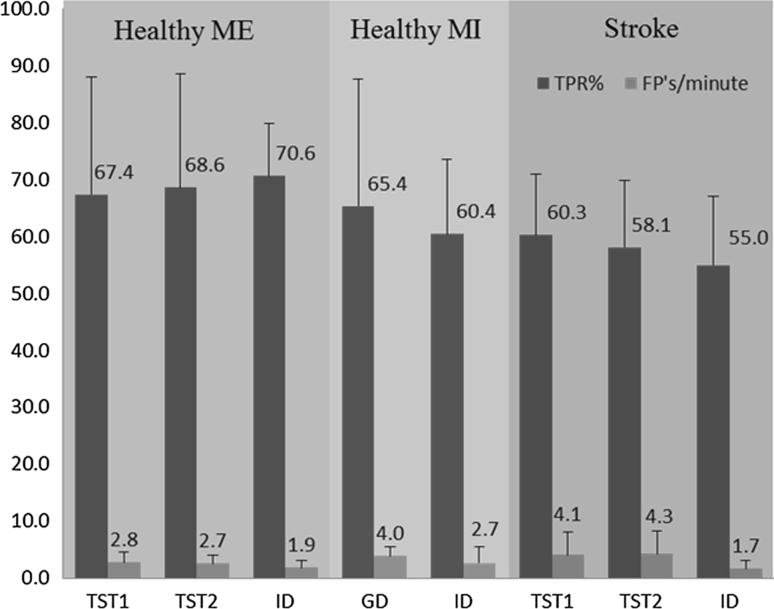
Performance for healthy and stroke subjects. Results from healthy motor execution (ME) and motor imagination (MI) (*N* = 20) and stroke patients (*N* = 5) testing data set with the TPR (%) (mean ± SD) and FPs per minute (mean ± SD). *TST1* represents all the runs of the subject being tested with global detector (GD), *TST2* are the identical testing run(s) as used for testing of the ID in motor execution/attempted task in healthy and stroke subjects, respectively

The TPR (%) and FPs per minute of the GD with TST1 and TST2 as test data set and the ID are presented in Fig. 2 for healthy subjects and stroke patients.

For ME, the average (across all healthy subjects) TPR obtained with the GD for the TST1 and TST2 data were 67 ± 21 and 69 ± 20 %, respectively, while the TPR of ID (71 ± 6 %) was slightly higher than that of the GD. The FPs per minute of the GD were 2.8 ± 1.7 (TST1) and 2.7 ± 1.3 (TST2), which was higher than those of the ID (1.9 ± 1.2). On the contrary, the average detection latency relative to the onset of movement obtained by the GD was −196 ± 162 ms (TST1) and −199 ± 147 ms (TST2), which was shorter than the latency of the ID (−85 ± 122 ms) (negative latencies indicate that the detection is leading the actual movement onset). The paired *t* test for the ME data did not reveal significant differences in TPR between TST1 and ID, nor between TST2 and ID (*P* > 0.05). For FP, paired *t* test revealed significant difference between GD and ID for both TST1 and TST2 (*P* < 0.05). Further, the latency was significantly better with the GD approach both when comparing TST1 and ID (*P* = 0.03) and TST2 and ID (*P* = 0.02).

For MI, the average TPR obtained with GD was 65 ± 22 % which was slightly greater than that of ID (60 ± 13 %) (Fig. 2). The FPs per minute were 4.0 ± 1.7 with GD and 2.7 ± 2.8 with ID. Paired *t* test did not find statistical difference in the performance indexes between GD and ID for the MI data.

For stroke patients, the average TPR obtained with GD were 60 ± 11 and 58 ± 12 % for TST1 and TST2, which was slightly higher than that of ID (55 ± 12 %). The FPs per minute of the GD were 4.1 ± 3.9 and 4.3 ± 4.1, for TST1 and TST2, respectively, which were greater than that of ID (1.7 ± 1.5). The average latency was 152 ± 239 and 162 ± 252 ms, for TST1 and TST2, respectively, with the GD, and 57 ± 140 with ID. In stroke patients, the morphology of MRCPs was different from that in healthy subjects as seen in Fig. 1b. Paired *t* test revealed no statistical difference (*P* > 0.05) for TPR, FP, and latencies in stroke patients across detector types.

## Discussion

To allow the use of BCI technology in clinical settings, the time for system setup calibration and optimization (training) has to be minimized. In this study, we introduced a method for the detection of movement intention from MRCPs that does not require an individual training on the subject but rather makes use of an ensemble dataset of previously collected signals from a population of subjects. In this way, the individual training phase is not needed. We compared this subject-independent detector with an individualized detector. The results indicated that the performance (TPR) with the proposed GD did not decline substantially as compared to that of the ID for data collected from a healthy and a stroke population. In addition, it was observed that the latency in detection was shorter in the healthy population when using the GD approach with respect to the ID. This result can be explained by the fact that the use of a MRCP template from a larger database may have reduced the high inter-subject and inter-session variability in the MRCP template. The stroke patient’s data showed the same trend, although not statistically significant which could be because of the smaller subject sample size and different pathological conditions of the individual patients.

There are only few studies which have used subject-independent training methods for BCIs. The works described in Refs. [11, 13] are based on sensorimotor rhythms (SMR) cue-based system for classification of two predefined movement imaginations (left vs. right hand movement). In both studies, a subject-independent training method exhibited a slight performance decrease as compared to that of individualized training approach. Contrary to these studies, our focus was on self-paced detection of movement intentions in healthy and stroke subjects.

With respect to other approaches for detecting movement imaginations (for e.g., based on beta rebound [15]), the proposed method provides the unique possibility of detecting/predicting motor intentions with short latency [14]. This is particularly useful for applications in neuromodulation, where the delay between the intention of action and the resulting feedback from the system is critical (within hundreds of ms from the movement onset) to induce changes in cortico-spinal excitability based on associative type of long-term potentiation (LTP), as shown in Refs. [11, 13]. Moreover, the detection accuracy and rate of false positives are in the same range as compared to that of [13] to induce changes in cortico-spinal excitability. Therefore, the proposed method can be potentially used to trigger peripheral muscle stimulation or any other assistive/restorative system for rehabilitation purposes. When interpreting the results on the MI task, it is necessary to consider that the motor task of button press of ~2 s after MI, for event marking, could potentially interfere with the MI detection.

The significant difference observed between latencies obtained from healthy ME task with GD and ID could be due to the fact that GD was built from a larger training data set as compared to ID. Moreover, the data of the present study (Fig. 1) show that the temporal and spatial profiles associated with the MRCPs are consistent across subjects, so that more data in the training set will result in template with greater SNR. Finally, the coefficient optimization process of OSF is signal (data) driven [14], which will provide better spatial optimization for better template and this will result in shorter latencies. It has to be noted that the detection latencies for stroke patients were positive which was likely due to the morphological differences in stroke and healthy MRCPs [9]. Generally because of stroke or other neurological disorders, sensorimotor integration is disturbed and this may result in delayed onset of reafferent potential, which is also evident from the MRCP shape after the onset in Fig. 1b. Because the lesion sites can be very different among stroke patients, potentially the GD approach might be better to build the detector from a group of subjects to counteract the inter-subject variability. There was indeed a trend for better performance with the GD approach in stroke patients.

In conclusion, this study demonstrates that it is possible to eliminate the conventional method of calibration for detection of intentional control during self-paced BCI’s based on single channel in healthy subjects and stroke patients.
